# Cold Atmospheric Plasma in the Treatment of Osteosarcoma

**DOI:** 10.3390/ijms18092004

**Published:** 2017-09-19

**Authors:** Denis Gümbel, Sander Bekeschus, Nadine Gelbrich, Matthias Napp, Axel Ekkernkamp, Axel Kramer, Matthias B. Stope

**Affiliations:** 1 Department of Trauma, Reconstructive Surgery and Rehabilitation Medicine, University Medicine Greifswald, Ferdinand-Sauerbruch-Straße, 17475 Greifswald, Germany; nadine.gelbrich@online.de (N.G.); matthias.napp@uni-greifswald.de (M.N.); traumato@uni-greifswald.de (A.E.); 2 Department of Trauma and Orthopaedic Surgery, BG Klinikum Unfallkrankenhaus Berlin gGmbH, Warener Str. 7, 12683 Berlin, Germany; 3 Leibniz-Institute for Plasma Science and Technology (INP Greifswald), ZIK *plasmatis*, Felix-Hausdorff-Str. 2, 17489 Greifswald, Germany; sander.bekeschus@inp-greifswald.de; 4 Department of Hygiene and Environmental Medicine, University Medicine Greifswald, Walther-Rathenau-Str. 49a, 17485 Greifswald, Germany; kramer@uni-greifswald.de; 5 Department of Urology, University Medicine Greifswald, Ferdinand-Sauerbruch-Straße, 17475 Greifswald, Germany; stopem@uni-greifswald.de

**Keywords:** apoptosis, cancer, CAP, osteosarcoma, plasma medicine, plasma oncology

## Abstract

Human osteosarcoma (OS) is the most common primary malignant bone tumor occurring most commonly in adolescents and young adults. Major improvements in disease-free survival have been achieved by implementing a combination therapy consisting of radical surgical resection of the tumor and systemic multi-agent chemotherapy. However, long-term survival remains poor, so novel targeted therapies to improve outcomes for patients with osteosarcoma remains an area of active research. This includes immunotherapy, photodynamic therapy, or treatment with nanoparticles. Cold atmospheric plasma (CAP), a highly reactive (partially) ionized physical state, has been shown to inherit a significant anticancer capacity, leading to a new field in medicine called “plasma oncology.” The current article summarizes the potential of CAP in the treatment of human OS and reviews the underlying molecular mode of action.

## 1. Introduction

**Figure 1:**
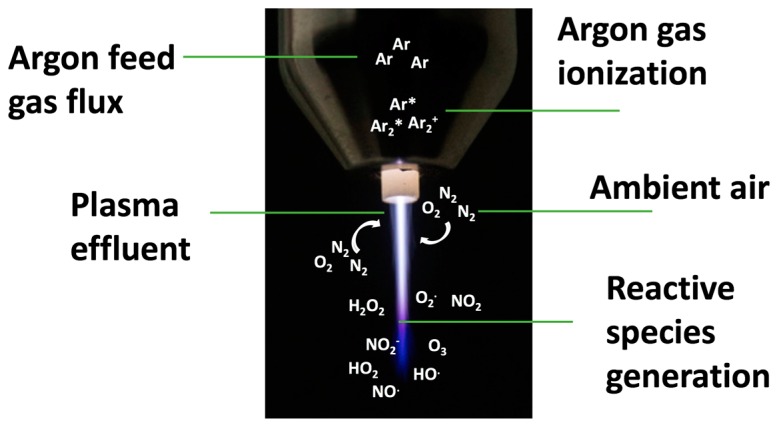
Principle of reactive species generation by cold physical plasma.

Cold atmospheric plasma (CAP) is a highly reactive (partially) ionized physical state containing a mixture of physical and biologically active agents. Depending on the plasma force, physical action is based on positive and negative ions, electrons, photons, and electromagnetic fields leading to the emission of visible ultraviolet (UV) or vacuum ultraviolet (VUV) radiation, and thermal effects. The probably most important components for biological effects, free radicals, include singlet oxygen (^1^O_2_), superoxide (O_2_^‒^), ozone (O_3_), hydroxyl radicals (^•^OH), nitrogen radicals (N_2_^•^), nitric oxide (^•^NO), nitrogen dioxide (^•^NO_2_), peroxynitrite (ONOO^‒^), hydrogen peroxide (H_2_O_2_), organic radicals, electrons, energetic ions, and charged particles (RO^•^, RO_2_^•^) (Figure 1) [1,2,3,4,5,6,7,8].

CAP operates at body temperature, making it feasible for a variety of medical applications, i.e., surface processing, inactivation of pathogens, or treatment of acute and chronic wounds [9,10]. Furthermore, plasma has been shown to inherit a significant anticancer capacity leading to a new field in medicine called “plasma oncology” [11]. Current cancer therapy aims at the complete eradication of cancer cells without affecting non-malignant tissue. However, complete surgical excision of tumor cells is challenged by microscopic tumor residues. CAP effects on cancer cells have been shown to involve the alteration of surface receptor functions, an activation of p53, induction of apoptosis or cell cycle arrest, and others. Together with its anticancer selectivity, CAP may therefore help to improve local tumor control when applied intraoperatively and further reduce the distance between the excised tumor and the surrounding healthy tissue necessary to achieve recurrence-free survival. The current review summarizes the potential of CAP in the treatment of human osteosarcoma (OS) and sheds light on the underlying molecular mode of action.

Figure 1 shows the atmospheric pressure plasma jet kINPen. A noble gas, in this case argon, is driven into the head of the jet, where a high-frequency electrode ionizes argon molecules. This argon plasma is then driven out into the vicinity where reactive argon molecules react with ambient-air resident oxygen and nitrogen molecules to form dozens of different reactive oxygen and nitrogen species. Major reactive moieties also dominate the coloring of the plasma effluent. This reactive species cocktail can be applied to cells and tissues to manipulate their redox signaling and ultimately induce cellular responses and killing.

### 1.1. Osteosarcoma (OS) Therapy Options

Human OS is the most common primary malignant bone tumor displaying a bimodal age distribution. It occurs most frequently in the metaphysis of long bones of children, adolescents, and adults over the age of 65. Approximately 10–20% of the patients already have metastases at the time of initial presentation [12]. OS is histologically characterized by sarcomatous stroma and the production of osteoid or premature bone tissue by malignant cells. With the advent of effective chemotherapy, survival of patients with OS has improved over the past decades. However, mortality remains high, and novel therapeutic approaches are urgently needed to improve outcome of patients suffering from this devastating disease. The combination of radical surgical resection of the tumor and systemic multi-agent chemotherapy is considered the backbone in OS therapy, but the heterogeneous nature of OS complicates treatment. Two cycles of chemotherapy with methotrexate, doxorubicin and cisplatin (MAP) are usually followed by limb salvage surgery rather than amputation, although optimal timing of chemotherapy remains elusive. Relative resistance of OS to radiation therapy (RT) leaves it as an option in cases where complete surgical resection of the tumor cannot be achieved. Local surgical control can be followed by adjuvant chemotherapy. The addition of Mifamurtide (MTP-PE) has been shown to improve the overall survival rate [13,14]. However, the search for novel targeted therapies to improve outcomes for patients with OS remains an area of active research including immunotherapy [15,16,17], photodynamic therapy [18], treatment with nanoparticles [19,20], and the combination of CAP and iron nanoparticles [21].

### 1.2. Cold Atmospheric Plasma (CAP) and Plasma Oncology

CAP applications have shown remarkable anticancer effects [22,23,24]. Inactivation and/or killing was observed in vitro in many tumor types, including melanoma [25,26,27], glioblastoma [28,29,30], pancreatic cancer [31], head and neck cancer [32,33,34], prostate cancer [35,36,37,38], colon cancer [39,40,41], lung cancer [42,43,44], leukemia [45,46,47], and gastric cancer [48]. Tumor damage and decline was observed in several in vivo models investigating, for example, pancreatic cancer [49,50], melanoma [51,52,53], ovarian cancer [54], breast cancer [55], and colon cancer [56]. It needs to be mentioned that many of these murine cancer models were not orthotopic, primarily because of technical deficits to reach with the plasma or plasma-treated liquid inside the animal body. Table 1 summarizes in which cancer systems CAP effects have been studied. Meanwhile, first case reports exist on beneficial plasma effects in cancer patients. The kINPen MED is accredited as medical device in Germany and the European Union for skin surface treatment and decontamination [57], although the classification as a device is at least controversial for wound treatment [58]. Final stage head and neck cancer patients often suffer from microbial infections [59]. These are difficult to eradicate due to tumor surface bleeding and irritation upon physical contact. Moreover, they cause strong odors, complicating social interaction, and thus palliation. Accordingly, head and neck tumors received gas-plasma treatment with the aim to decrease microbial burden. Unexpectedly, some tumors responded to plasma treatment [60], and cancer cell apoptosis was identified [31,61,62,63]. First reports on the use of CAP for tumor removal dates back to 1989 [64], followed by successful ablation of non-neoplastic Barrett’s mucosa [65] and neoplastic diseases. Recently, the first patient worldwide profited long lasting from plasma tumor therapy [66].

### 1.3. CAP Devices and General Biological Impact

There are two groups of cold plasma sources. The majority are experimental sources (please refer to other reviews about information on them [67,68,69]). The second group are accredited devices. In Europe, so far four devices are available as medical devices. As a first step, the technical standard DIN SPEC 91315:2014-06 was developed, which characterizes the basic physical and technical performance parameters of CAP sources to be used for bio-medical or biological experiments and for further development to become medically applicable plasma sources [70]. Three CAP sources received accreditation through clinical observations/studies [59], but none of them is licensed for oncological applications yet. All CAP sources expel a plethora of reactive species [71]. These have been implicated as active agents in oncotherapy [72]. For a long time, it was assumed that cancer cells suffer oxidative stress per se, making them more vulnerable to additional exposure to oxidants [73]. Accordingly, anticancer effects of plasma are often referred to as being selective to tumor over non-tumor cells [74,75,76]. However, this view is challenged by the understanding that oxidative damage is also mediated via redox signaling [77], i.e., its translation to cell death can in principle be counter-regulated in cancer cells [78]. A number of studies comparing several cell lines corroborate this notion, showing that the sensitivity of a given cell type or cell line seems not always to depend on its tumorigenic potential [79,80,81]. Yet, without a doubt plasma effects are primarily mediated via reactive species [82,83,84]. Some studies highlighted a possible role of, for instance, UV/VUV radiation [85,86,87] or electrical (field) effects [88], but clear evidence of any major contribution similar to reactive species is lacking.

## 2. CAP Effects on OS Cells

Due to aggressive chemotherapy regimen, OS therapy is frequently attended by the risk of treatment toxicity, and there is thus an unmet clinical need for novel therapeutic strategies [12]. The multifunctional impact of CAP on cancer cell response and survival has been demonstrated in several solid cancer entities. Therefore, CAP treatment might be a promising alternative in future OS therapy concepts. However, the underlying molecular mechanisms are not completely understood.

### 2.1. CAP-Induced Redox Effects and Redox Signaling

Cancer cells display weaker antioxidant mechanisms compared to normal cells [33,89]. In prostate cancer cells, redox detoxification capacity is altered by decreased intracellular glutathione (GSH) levels compared to non-malignant prostate cells [36]. Thus, they can be attacked selectively by a CAP-induced increase of extracellular and intracellular reactive oxygen species (ROS) and reactive nitrogen species (RNS) (reactive oxygen and nitrogen species, RONS). By utilizing a preclinical in vitro OS model system consisting of the permanent OS cell lines U2-OS and MNNG/HOS, a singular CAP treatment of 10 s of tumor cells in suspension was sufficient for the significant inhibition of cellular growth [90]. Notably, this antiproliferative effect was neutralized by supplementation of *N*-acetylcysteine (NAC), a low-molecular weight substance related to the glutathione-dependent cellular redox system [91]. Transmembrane diffusion of extracellular ROS such as H_2_O_2_ plays a critical role in intracellular ROS increase. Macromolecules below a radius of 6.5 nm were able to enter HeLa cells following CAP treatment and consecutively induced temporary cell permeabilization [92,93]. Aquaporin (AQP) expression has been shown to be upregulated in tumors, increasing H_2_O_2_ uptake in malignant compared to non-malignant cells explaining diverse responses of cancer cells following CAP treatment [94,95]. Furthermore, increased mitochondrial transmembrane permeability via a CAP induced depolarization of the mitochondrial membrane potential results in the release of proapoptotic factors [96]. After absorption into the cell, NAC can serve as a substrate for the biosynthesis of GSH. GSH itself as well as GSH-dependent enzymes are essential factors in the GSH-dependent redox homeostasis system and have been demonstrated to play a crucial role in chemoresistance mechanisms of OS cells representing a detoxification pathway [97]. For this reason, and due to the composition of CAP containing reactive species, an involvement of cellular redox processes appears most likely. For instance, it has been shown that CAP treatment leads to lipid peroxidation and mitochondrial membrane potential decrease [98,99,100]. In cell culture, CAP treatment triggers the de novo formation of hydrogen peroxide (H_2_O_2_) in the cell culture medium, with a production rate depending on the composition of the medium used [101]. Subsequently, H_2_O_2_ may overcome the cytoplasmic membrane elevating the intracellular H_2_O_2_ level. Furthermore, CAP induced redox stress activates cellular detoxification systems and provokes the enzymatic formation of intermediary H_2_O_2_ in the cells [102]. Generally, processes of redox signaling cascades and detoxification pathways are activated immediately.

In OS cells, CAP specifically controls members of an antioxidant enzyme family, namely peroxiredoxins (Prx). After CAP treatment, cytosolic Prx isoforms Prx-1 and Prx-2, but not the mitochondria-specific isoform Prx-3, were reduced to the non-catalytic monomeric form [91]. Moreover, CAP activated the secretion of Prx-2, but not Prx-1 and Prx-3, into the extracellular space. The cellular functionality of cell-free Prx-2, however, is unclear. Beside an inactivation of Prx-1 and Prx-2 enzymes by protein's redox status, CAP additionally affects Prx-mediated signal transduction controlling cell growth arrest, apoptosis and proliferation [77]. These findings point to a role for redox-specific signaling pathways in CAP induced proliferation control.

### 2.2. CAP-Induced Apoptosis

The induction of programmed cell death (apoptosis) by cytostatic agents is a common mode of action in cancer treatment and in OS chemotherapy in particular [103,104,105] and mitochondria act as the major regulator of apoptosis [96]. Triggered by specific signaling cascades, apoptosis results in cellular degradation and cell death without liberation of degradation products and critical biomolecules, which may induce systemic processes of inflammation or immune response [106]. The induction of apoptotic effects in cancer cells has been shown in several solid tumors including carcinoma of prostate, breast, and pancreas [21,31,36].

In human cervical cancer HeLa cells, CAP induced various intracellular and extracellular signals by oxidative stress converge in mitochondria, increasing their transmembrane potential and promoting the release of pro-apoptotic factors including cytochrome c. This process is regulated by the Bcl-2 protein family and ultimately leads to the activation of the caspase cascade [96]. In a melanoma cell line, pro-apoptotic changes such as Rad17 and tumor suppressor p53 phosphorylation, cytochrome c release, and caspase-3 activation were initiated by CAP [107].

Using an in vitro cell culture model, induction of apoptotic events in CAP-treated OS cells has been demonstrated based on diverse methods and measured at different stages of the apoptotic cascade [90,108]. CAP-induced anti-proliferative efficacy in OS cells was accompanied by the induction and phospho-activation of the tumor suppressor protein p53 [90]. This so-called “guardian of the genome” can block entry into the cell cycle and can induce apoptosis via both intrinsic and extrinsic apoptotic pathways. In anticancer therapy, the increase of p53 activity is frequently part of treatment-induced efficacy and may initiate the apoptotic effects in CAP treated OS cells.

Beside energy-dependent signal transduction pathways and enzymes like nucleases and proteases, apoptosis is also characterized by stage-dependent morphological alterations, e.g., cell shrinking, chromatin condensation, nuclear deformation, and finally the formation of small apoptotic bodies as one of the last stages before final degradation [109]. In U2-OS and MNNG/HOS OS cell lines, CAP treatment activated the apoptosis-specific proteases caspase-3 and caspase-7 [unpublished data]. Later in the apoptotic cascade, cell shrinking has been demonstrated by fluorescence dye stained OS cell nuclei [110]. Microscopic analysis revealed decreased nuclei measured by a reduced nuclear area and perimeter. Furthermore, the condensation of chromosomal DNA was detected, expressed as the intensified total fluorescence signal within the nuclei [unpublished data]. The apoptosis-specific degradation of the chromosomes could be demonstrated by performing TUNEL assay as well as comet assay [unpublished data].

Another mechanism is the alteration of the cell cycle. CAP increased the percentage of apoptotic tumor cells by blocking the cell cycle at the G2/M checkpoint, and this effect was mediated by reduced intracellular cyclin B1 and cyclin-dependent kinase 1 (Cdc2), increased p53 and cyclin-dependent kinase inhibitor 1 (p21), and increased Bcl-2-like protein 4 (Bax)/B cell lymphoma 2 (Bcl-2) ratio [111].

### 2.3. CAP-Induced Gene Expression and Epigenetic Changes

In addition to direct effects on tumor cells as aforementioned, CAP interferes with a number of other processes that indirectly influence tumor cell growth. For instance, CAP treatment of physiological solutions and cell culture media have been shown to exert antiproliferative effects on tumor cells [112].

Oxidative stress induced by CAP can modify the expression of nearly 3000 genes encoding structural proteins and inflammatory mediators, such as growth factors, cytokines [113], interleukins and members of the tumor necrosis factor (TNF) superfamily. Cytokine and chemokine expression has been demonstrated to be targeted by CAP treatment followed by the modulation of systemic processes, e.g., inflammation and immune response [114,115,116].

Regarding OS tumor biology and particularly future OS therapy concepts, a transcriptomic profiling of CAP-treated cells utilizing a cytokine and chemokine-specific DNA array was performed. Altogether, a total of 84 cytokines and chemokines were analyzed by quantitative polymerase chain reaction (qPCR). Here, the expression rate of several factors was significantly modulated after CAP treatment of OS cell lines U2-OS and MNNG/HOS. Of the 84 investigated cytokines, 9 (U2-OS) and 8 (MNNG/HOS) factors, respectively, were differentially regulated compared to control approaches [unpublished data]. Within these factors, 3 chemokines (C5, CCL5, CXCL1) but primarily 5 interleukins (IL-1A, IL-1B, IL-18, IL-22, IL-23A), and 7 growth factors (CNTF, CSF1, CSF3, MSTN, NODAL, TGFB2, THPO) were significantly induced in the presence of CAP. Notably, only vascular endothelial growth factor (VEGFA), an inductor of angiogenesis, was suppressed by CAP application.

Taken together, the presented examination suggests that the modulation of cytokines and chemokines after CAP treatment interferes with proliferation, and chemotaxis and may affect tumor angiogenesis, invasion, and metastasis development.

**Figure 2:**
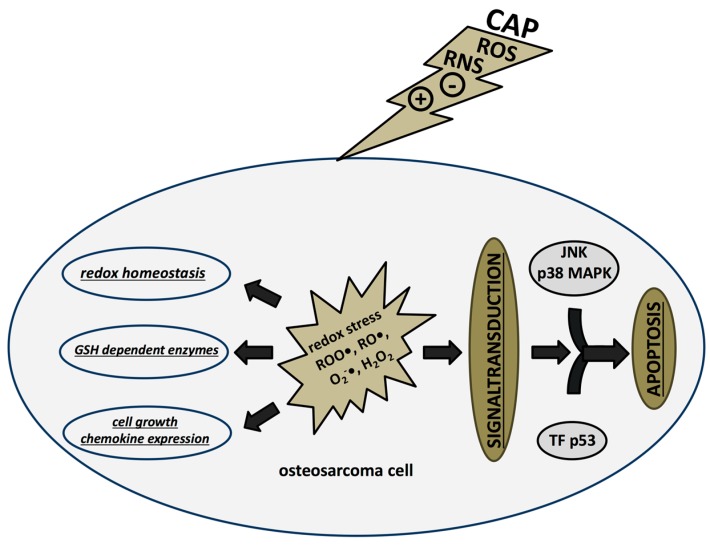
CAP effects on osteosarcoma cells. CAP, cold atmospheric plasma; reactive oxygen species (ROS), reactive nitrogen species (RNS), glutathione (GSH), transcription factors (TF), c-Jun N-terminal kinases (JNK).

In a fibroblast culture and in a wound healing mouse model CAP increased the expression of type I collagen and genes encoding proteins involved in wound healing processes (interleukin 6 [IL-6], IL-8, chemokine [C–C motif] ligand 2 [CCL2], transforming growth factor β1 [TGF-β1], TGF-β2, CD40 ligand, chemokine [C–X–C motif] ligand 1 [CXCL1], interleukin 1 receptor antagonist [IL-1RA], and plasminogen activator inhibitor-1 [PAI-1]) without affecting cellular migration, proliferation, and apoptosis [117]. In the context of psoriasis CAP has been shown to induce downregulation of IL-12 and upregulation of IL-1β, IL-6, IL-8, IL-10, tumor necrosis factor α (TNF-α), interferon gamma (IFN-γ), and vascular endothelial growth factor (VEGF) mRNAs in human keratinocytes [118]. Park et al., (2015) first demonstrated changes in DNA methylation pattern in a breast cancer cell line expressing the estrogen receptor (MCF-7) and one that does not express it (MDA-MB-231). Epigenetic modifications were more extensive in MCF-7 cells, affecting the promoter region of genes related to “cell mobility”, “connective tissue function and development”, “motility development”, “cell–cell communication and cell–cell interaction”, and “cell survival and cell death” (Figure 2) [119].

CAP-induced intracellular and extracellular redox stress impairs OS redox homeostasis, GSH dependent enzymes, and cell growth and alters chemokine expression patterns. Direct effects on signal transduction cascades leads to apoptosis.

## 3. Clinical Prospects and Conclusions

First in vitro studies suggest cold plasma to be effective against osteosarcoma. Animal models are needed to stratify this conclusion in more biologically relevant systems. Plasma is regarded as potential adjuvant therapy. Its therapeutic efficacy should therefore be assessed in combination with current drugs used for OS therapy in vitro and in vivo. Furthermore, prior to the clinical application of CAP, several technical parameters need to be studied in more detail, including penetration depth, optimal dosage, and repetitive applications. Also, recent evidence suggests a potential synergy of nanoparticles in combination with CAP that leads to a maximized targeted efficacy [120]. At the same time, the killing efficacy of plasma should be tested on chemoresistant cancer cells. Vice versa, repetitive applications of plasma on these cancers in vitro will show the presence or absence of adaption processes to frequent oxidative challenge by the tumor cells. Once validated for OS therapy in pre-clinical models, plasma therapies need to be embedded within specific cancer treatment modalities. This could be, for example, as an adjuvant tool during chemotherapy and resection, as a potent killer of micrometastases outside the surgically removed bulk tumor, or during palliative care.

## Figures and Tables

**Table 1:** Cancer systems used for investigating cold atmospheric plasma (CAP) effects.

Species	Tumor	Cell Line
human	non-small cell lung cancer (NSCLC)	MR65, SW900
human	hepatocellular carcinoma	HepG2, BEL-7402
human	melanoma cells	A2058, G361, SK-MEL-28
human	cervical cancer	HeLa
human	colon carcinoma	COLO320DM, HCT-116, SW480, LoVo
mouse	melanoma cells	B16-F10, 1205Lu, Mel Juso, Mel Ei, Mel Ho, Mel Im, Mel Ju, HTZ19, A375
human	breast cancer	MCF-7, MDA-MB-231
human	glioblastoma cells	U87, T98G, LN18, LN229
human	bladder cancer cells	SCaBER
mouse	lung carcinoma cells	TC-1
human	acute lymphoblastic leukaemia cells	CCRF-CEM
human	pancreatic cancer cells	MIA PaCa2-luc, Colo-357, PaTu8988T
human	ovarian cancer cells	SKOV-3, HRA
mouse	pancreatic cancer cells	6606PDA
human	acute monocytic leukaemia cells	THP-1
human	skin cancer	PAM212
human	lung cancer	H460, A549
mouse	neuroblastoma	Neuro2a
human	head and neck squamous cell carcinoma cells	JHU-022, JHU-028, JHU-029, SCC25, FaDu, OSC 19
human	prostate cancer	LNCaP, BPH-1, PC-3
human	oral squamous cell carcinoma cells	HSC-2, SCC-15
human	multiple myeloma cells	RPMI8226, LP-1
human	lymphoma	U937
human	osteosarcoma	U2-OS, MNNG, SaOS-2
